# Migration Status and Prevalence of Chronic Diseases in Kerala State, India

**DOI:** 10.1155/2013/431818

**Published:** 2013-12-10

**Authors:** Safraj Shahul Hameed, Vellapallil Raman Kutty, Krishnapillai Vijayakumar, Ajayan Kamalasanan

**Affiliations:** Health Action by People, TC 1/1706, Chemmanam, Navarangam Lane, Opp. 3rd Men's Hostel, Medical College PO, Trivandrum, Kerala 695 011, India

## Abstract

*Aim*. To study the relationship between a personal history of migration and prevalence of chronic diseases and risk factors in a rural population. *Method*. Cross sectional survey data from PROLIFE, a cohort study involving the long time follow-up of the residents of an administrative unit in Kerala, India, was used. Pre-tested questionnaire was administered to 78,173 adult residents. Information on physician diagnosed diabetes, hypertension, and cardiac diseases and lifestyle attributes like physical activity, habits, and migration was captured. *Results*. Subjects with a history of migration had a higher prevalence of chronic disease when compared with those with no history of migration. Diabetes (19.6% versus 4.1%), hypertension (18.8% versus 6.6%), and cardiac complaints (8.6% versus 4.1%) are more prevalent among those with history of migration of over 5 years. After adjustment for age, gender, and education, we found that chronic diseases are higher among persons with a history of migration (OR 2.2, 95% CI: 2.1–2.3). Age-specific increases in prevalence of chronic diseases are also substantially higher among migrants. *Conclusion*. People with a history of migration have a higher prevalence of chronic diseases and risk factors.

## 1. Introduction

Migration is a process of social change during which people move from one cultural setting to another in order to settle for a longer period of time or permanently [1]. Though migration has been occurring since the beginning of time, the advent of the era of globalization has resulted in the increased movement of people across and within national boundaries for various reasons. The social, cultural, financial, and other aspects of migration have been the focus of much research and many new policies initiated as a result. The relationship between migration and health has also been well explored. However, there is a lack of consistency among studies of migration and health with results indicating both higher and lower prevalence of diseases among migrants in relation to the residents [1–9]. The health status of return migrants, migrants, who have returned to their place of origin, has not been the focus of much research. A study from Mexico had previously reported on the disadvantages faced by the return migrants [10]. More research is needed in this area as the health status of the return migrants may be much different from that of the resident community. In this exploratory study, we address this information gap by studying a rural community in the south Indian state of Kerala which has a long history of migration.

The state of Kerala with a population of 34 million enjoys one of the best health indicators in India with an infant mortality rate of 12 and life expectancy at birth of 74 years [11]. These levels are far better than the other Indian states and are quite close to those of developed countries. Kerala has also made significant progress economically with recent economic indicators showing that Kerala is one of the Indian states with the least reported levels of poverty [12]. A significant proportion of the total receipts of Kerala are contributed by remittances from emigrants. The remittance from these migrants formed 28% of the state revenue receipts [13]. As of 2007, 1.85 million Keralites were living abroad; of theses, 89% were residing in the Persian gulf countries [14]. Surveys also indicate that one out of four households in Kerala had at least one resident abroad. An interesting feature of migration in Kerala is that a good proportion of the migrants return home after working for various periods in foreign countries. As in 2007, there were 890,000 return emigrants, roughly one return emigrant for every two living abroad [14].

Another thing to note is the very high prevalence of chronic diseases in Kerala, with the burden of deaths from cardiovascular diseases now exceeding that of industrialized countries [15]. Our own previous study has shown that the prevalence of self-reported diabetes was higher among the richer socioeconomic groups than the poorest group [16].

With the Kerala society at an advanced stage of demographic and epidemiological transition, research which focuses on this change is vital. It was with this view that the population registry for life style diseases (PROLIFE) study was initiated [17]. The initial goal was to assemble a cohort to study the epidemiology of chronic diseases in the selected rural community and to estimate the burden of risk factors. Here we study the influence of migration on the health status of individuals by comparing the health status of return migrants with that of the residents who had never worked abroad. The present study explores the prevalence of selected chronic disease/risk factors among return migrants in comparison to age and sex matched local residents. We also explore the relationship between the duration of migration and prevalence of risk factors.

## 2. Methods

This is a cross sectional survey involving the adult subjects of the PROLIFE study. PROLIFE is a prospective cohort study initiated in 2001 involving the long time follow-up of the residents of Varkala “Integrated Child Development Services” (ICDS) block in Trivandrum district, Kerala. All residents were enrolled in the study; 1, 61,942 subjects living in 33,379 households were included in the baseline survey. Subjects aged 20 years or more were enrolled into an adult cohort. Separate adult questionnaires were administered to 78,173 subjects (33,978 men and 44,195 women) who were 20 years as of January 1, 2002. This constituted 80.4% of eligible members. The shortfall was primarily on account of some subjects being absent from home on three consecutive visits. Other than deaths, migration would be the main reason for loss to follow-up. As of December 31, 2004, 721 subjects moved out of the study area and were lost to followup. This constitutes <0.01% of the subjects, over a period of 42 months. Further details of the study are provided elsewhere [17].

105 female healthly workers involved in routine mother and child care served as field investigators. A team of physician epidemiologists trained the workers in survey methods and in the recognition of common disease conditions. The workers were also trained on collecting cause of death information using a structured questionnaire.

The baseline survey collected household level information capturing demographic, educational, and a host of socio economic attributes. Additionally, information on deaths and births that occurred during the year previous to the survey was gathered. A pretested questionnaire was administered to every adult subject, (aged 20 years or more on 1 January, 2001). This questionnaire captured information on diagnosed diabetes, hypertension, and cardiac diseases, in addition to lifestyle attributes like physical activity, habits and migration history. Diabetes, hypertension and cardiac diseases were reported by the subjects as diagnosed by a physician on the basis of clinical and biochemical parameters. Details of habits such as tobacco usage and alcohol consumption were collected. Information on the duration and destination of migration and type of employment during that time was ascertained. Resource limitations prevented us from capturing dietary details and anthropological measurements in the present survey.

The objectives of the study were explained to the head of each household who had the freedom to refuse. Informed oral consent was obtained from every respondent and the study was overseen by an independent ethics committee.

Statistical analysis for differences between nonmigrants and migrants was carried out. Age-specific prevalence of diabetes, hypertension, and history of cardiac complaints among males and females was calculated. We analyzed data for possible relationship between the duration of migration and observed prevalence of the diseases by doing Mantel test for trend. Logistic regression analysis adjusting for confounders such as gender, education, and age was carried out to calculate the adjusted odds ratio.

## 3. Results

We interviewed 78173 subjects to assess their migration status. Of these, 8332 persons reported living outside Kerala for more than 1 year. Information was not provided by 603 subjects.

Table 1 provides the salient features of the population and compares the characteristics of subjects with and without history of migration. The majority of subjects who reported a history of living outside Kerala were male (86.3%). The mean age of subjects with a history of migration was higher than that of subjects with no history of migration (45.7 versus 41.4 years). Return migrants had better educational levels with only 15.6% of subjects reporting low or less than 5 years of education, whereas 28% of subjects with no history of migration reported their educational status as low. Prevalence of risk factors such as smoking (49.7% versus 17.6%) and alcohol consumption (34.6% versus 11.6%) were higher among return migrants. Similarly, return migrants reported higher prevalence of diabetes (14.1% versus 6.0%), cardiac conditions (6.6% versus 3.7%), and hypertension (14.7% versus 9.9%).

**Figure 1:**
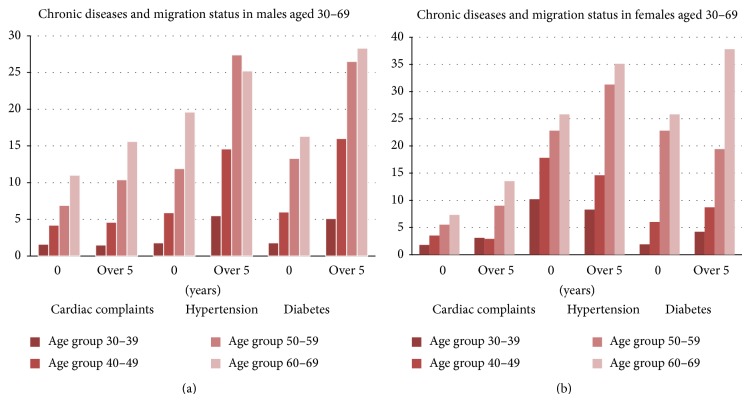


Age-specific prevalence of diabetes, hypertension and history of cardiac complaints among males and females aged 30–69 with a history of living outside Kerala for more than 5 years and those with no history of migration is provided in Figures 1(a) and 1(b).

Figure 1(a) presents data on age-specific prevalence of the diseases among males with a history of migration and nonmigrants. Diabetes, hypertension, and cardiac complaints are more prevalent among those with duration of migration of over 5 years when compared with those with no history of migration. The age-specific increase in prevalence is substantially higher among migrants for diabetes and hypertension though the trend is unmistakable even for cardiac diseases. The same trend is seen for women aged 30–69 as seen in Figure 1(b).

Table 2 provides the odds ratio for the presence of physician diagnosed chronic diseases (diabetes, hypertension, and cardiac conditions). After adjustment for age, gender, and education, subjects with a history of migration had higher odds of having chronic diseases (OR 2.2, 95% CI 2.1–2.3).

The conditions showed an increase in prevalence with the number of years of migration. The highest prevalence of all risk factors was noted in persons with a history of migration of more than 10 years. Statistical analysis for differences between nonmigrants and migrants reveals high significance. We analyzed that data for possible relationship between the duration migration and observed prevalence of the diseases by doing Mantel test for trend.  Table 3 shows that there is a clear trend of increased prevalence in relation to duration of migration.

## 4. Discussion

In our study, residents with a history of migration reported a higher prevalence of cardiac diseases, hypertension, and diabetes. The prevalence increased with the duration of migration with those residents who lived outside Kerala for more than 5 years showing the highest prevalence.

Our finding that diabetes, hypertension, and cardiac diseases are substantially higher in migrants corroborates some of the previous publications in this regard. Like similar studies, our study also has certain limitations. Prevalence of chronic conditions has been estimated based on the self reporting of physician diagnosed conditions. This could result in an under estimation of the actual community prevalence. Also, our analysis is restricted to subjects who are currently residing in the study area. Since these diseases tend to increase with age, the higher prevalence may be attributed to the age effect and not to the fact of a history of migration. However, logistic regression analysis showed that, after adjustment for age and gender, subjects with a history of migration had a higher prevalence of chronic diseases. The data on age-specific prevalence also disproves such an explanation; between 30 and 70 years the prevalence is significantly higher among migrants in all four age groups. We can therefore conclude that the most important contributory factor to the observed increase in prevalence is history of migration.

Our analysis on the prevalence of 2 significant risk factors namely smoking and alcohol consumption for noncommunicable diseases in our study subjects clearly establishes that the prevalence for both risk factors is more than double that in those with a history of migration. Also to be noted that there are the unusual high rates of drinking and smoking among women with a history of migration. This phenomenon requires further study and we believe that in the context of this study we explain the high prevalence of chronic diseases among migrant women in this rural community.

Worldwide, studies on the health conditions of migrants show a mixed picture. Infectious diseases like TB have been shown to be high among migrants than the resident population [18, 19]. Chronic diseases like diabetes and stroke have also been shown to be high among migrants [6, 20, 21]. Higher incidence of mental disease and alcohol and drug abuse was also seen among migrants [22, 23]. Migrants from the Indian subcontinent in many cases had higher incidence of disease than migrants from elsewhere [6, 20, 24, 25].

However, some studies have shown that the mortality rate of migrants though higher than that of the residents are lower than that of their country of origin [26–28]. This apparent paradox has been explained as due to reasons such as the “healthy migrant effect,” where by the persons who migrate are healthier than the nonmigrants and the “salmon effect,” where by elderly migrants return to their country of origin to die [29–32].

It has been observed that the urbanization process associated with migration leads to the availability and abundance of calorie-dense/low-fiber foods and the adoption of sedentary lifestyles. This leads to increased risks of morbidity and mortality from diet and lifestyle-related chronic diseases. A recent report which focused on return migrants reported that the health of returning migrants is impacted by the cumulative exposure to social determinants and risk factors of health during the migration process, during the return movement, and following return [33].

The remittances from migrants constitute a major part of the revenue for the state of Kerala and the economy of Kerala is much dependent on continuing remittances from the migrants. Migrants make a significant contribution to the prosperity of the state. However, this prosperity appears to come with a heavy personal price. The government of Kerala has initiated various programs which focus on the welfare of the nonresident Keralites and has a dedicated department which looks after affairs of the nonresident Keralites called NORKA [34]. However, at present, most of the programmes concentrate on providing financial, labor, and rehabilitation assistance to return migrants. The inclusion of health as a major component in NORKA ROOTS programme and similar programmes can have a significant effect on the health of migrants and return migrants. It is imperative that the state initiates programs that target the health and wellbeing of this very important segment of the population.

## Figures and Tables

**Table 1:** Baseline characteristics of study subjects.

Characteristic	Migrant (%)	Nonmigrant (%)
Subjects	8332	69211
Male	7190 (86.3)	26503 (38.3)
Female	1142 (13.7)	42708 (61.7)
Mean age (SD)	45.7 (13.4)	41.4 (16.3)
Education		
Low	1273 (15.6)	18673 (28.0)
Intermediate	6301 (77.0)	41545 (62.4)
High	611 (7.5)	6405 (9.6)

Risk factors and chronic diseases		
Smoking		
Male	4014 (56.1)	11670 (44.2)
Female	107 (9.5)	454 (1.1)
Alcohol		
Male	2787 (38.9)	7719 (29.3)
Female	81 (7.2)	232 (0.5)
Diabetes		
Male	1080 (15.1)	1557 (5.9)
Female	115 (10.1)	2601 (6.0)
Cardiac conditions		
Male	501 (7.1)	1093 (4.2)
Female	50 (4.4)	1431 (3.4)
Hypertension		
Male	1029 (14.4)	1742 (6.6)
Female	186 (16.4)	5080 (12)

**Table 2:** Adjusted Odds Ratio (OR) for presence of diagnosed chronic diseases/risk factor.

	Odds ratio (95% CI)	*P* value
**Migration history**	2.19 (2.06–2.33)	<0.001
Advanced age	1.06 (1.06–1.07)	<0.001
Female sex	0.77 (0.70–0.77)	<0.001
Low education	1.14 (1.05–1.25)	<0.001

Bold font refers to migration history the primary outcome of interest.

**Table 3:** Duration of migration and prevalence of selected chronic diseases.

	% Never lived outside	% Lived outside 1–5 years	% Lived outside more than 5 years	χ^2^ (mantel test for trend)	*P* value
CHD	4.1	4.7	8.6	251.75	<0.001
Hypertension	6.6	8.3	18.8	205.93	<0.001
Diabetes	5.9	8.4	19.6	991.19	<0.001
